# Quantifying the burden of caregiving in Duchenne muscular dystrophy

**DOI:** 10.1007/s00415-016-8080-9

**Published:** 2016-03-10

**Authors:** Erik Landfeldt, Peter Lindgren, Christopher F. Bell, Michela Guglieri, Volker Straub, Hanns Lochmüller, Katharine Bushby

**Affiliations:** Institute of Environmental Medicine, Karolinska Institutet, Nobels väg 13, SE-17177 Stockholm, Sweden; OptumInsight, Klarabergsviadukten 90, SE-11164 Stockholm, Sweden; Medical Management Centre, Department of Learning, Informatics, Management and Ethics, Karolinska Institutet, Tomtebodavägen 18A, SE-17177 Stockholm, Sweden; GlaxoSmithKline, 5 Moore Drive, PO Box 13398, Research Triangle Park, Durham, NC 27709-3398 USA; Newcastle University John Walton Muscular Dystrophy Research Centre and MRC Centre for Neuromuscular Diseases, Institute of Genetic Medicine, International Centre for Life, Central Parkway, Newcastle upon Tyne, NE1 3BZ UK

**Keywords:** Caregiver burden, Quality of life, Utilities, Informal care, Neuromuscular

## Abstract

Duchenne muscular dystrophy (DMD) is a rare pediatric neuromuscular disease associated with progressive muscle degeneration and extensive care needs. Our objective was to estimate the caregiver burden associated with DMD. We made cross-sectional assessments of caregiver health-related quality of life (HRQL) and burden using the EuroQol EQ-5D, a Visual Analogue Scale (VAS), the SF-12 Health Survey, and the Zarit Caregiver Burden Interview (ZBI) administered online. Results were stratified by disease stage (early/late ambulatory/non-ambulatory) and caregivers’ rating of patients’ health and mental status. In total, caregivers to 770 patients participated. Mean EQ-5D utility ranged between 0.85 (95 % CI 0.82–0.88) and 0.77 (0.74–0.80) across ambulatory classes and 0.88 (0.85–0.90) and 0.57 (0.39–0.74) across caregivers’ rating of patients’ health and mental status. Mean VAS score was 0.74 (0.73–0.75), mean SF-12 Mental Health Component Summary score 44 (43–45), and mean ZBI score 29 (28–30). Anxiety and depression, recorded in up to 70 % of caregivers depending on patients’ health and mental status, was significantly associated with annual household cost burden (>$5000 vs. <$1000, odds ratio 1.76, 95 % CI 1.18–2.63) and hours of leisure time devoted to informal care per week (25–50 vs. <25 h 2.01, 1.37–2.94; >50 vs. <25 h 3.35, 2.32–4.83) (*p* < 0.007). We show that caring for a person with DMD can be associated with a substantial burden and impaired HRQL. Our findings suggest that caregivers to patients with DMD should be screened for depression and emphasize the need for a holistic approach to family mental health in the context of chronic childhood disease.

**Electronic supplementary material:**

The online version of this article (doi:10.1007/s00415-016-8080-9) contains supplementary material, which is available to authorized users.

## Introduction

Throughout the Western world, including the US, Australia, Japan, and the European Union, long-term care of disabled or chronically ill patients is predominately provided at home by untrained, unpaid family members [1–4]. For many caregivers, assisting their spouse, parent, offspring, or other relative in their day-to-day life may be a positive experience, but can at times also be tremendously challenging. Numerous studies have shown that the provision of informal care is associated with serious adverse health effects for the caregiver, including anxiety and depression, impaired immune system function, and coronary heart disease, as well as social isolation, financial deprivation, and even premature death [1, 5–7]. Existing research, however, focus on the burden on caregivers to elderly patients, predominantly with dementia, with less attention devoted to genetic pediatric conditions, including Duchenne muscular dystrophy (DMD).

DMD is a rare, X-linked, neuromuscular disease characterized by progressive muscle weakening, diminishing functional ability, and serious multisystem complications, with a mean life expectancy of 25 years [8–10]. As a result of the devastating disease progression, patients inevitably transition towards a state of total dependency, requiring wheelchairs for mobility from their early teens and ventilation support for survival in more advanced stages of the disease. Many patients with DMD also suffer from mental health comorbidities, such as autism spectrum disorder (ASD) and obsessive–compulsive disorder (OCD). The complexity of the disease necessitates multidisciplinary management including regular visits to neuromuscular, cardiac, and respiratory specialists, physiotherapists, and other healthcare practitioners [8, 9].

We have previously reported that caring for a child with DMD is both time-consuming and financially burdensome [11]. Many DMD caregivers terminate their employment or reduce their working hours to find the time needed to care for their sons, and those who do continue to work have markedly impaired productivity with high levels of absenteeism. In addition to forgone income, depending on national policies, affected households also carry substantial costs associated with, e.g., insurance premiums and co-payments for healthcare.

The caregiver burden has been studied in DMD only in small samples from a single clinic or country, in combination with other muscle dystrophies, and/or without stratifying results by disease stage or patient health status [12–22]. The objective of this multinational study was to complement our previous data on the objective caregiver burden in DMD with estimates of the subjective burden: that is, the impact on health-related quality of life (HRQL). A specific aim was to investigate mental distress among caregivers.

## Methods

### Participants and procedures

Caregivers to patients with DMD were recruited as part of a multinational, cross-sectional, observational study for which details and results have been previously reported [11, 23]. In summary, patients with DMD from Germany, Italy, the UK, and the US were recruited through national DMD registries which form part of the global TREAT-NMD network [24]. To be eligible, patients were required to fulfill the following criteria: (1) male, (2) DMD diagnosis, and (3) age ≥5 years. Caregivers to eligible patients were invited to complete a questionnaire online. All participants provided informed consent and study approval was granted from Ludwig-Maximilians-Universität München (Germany), Comitato Etico IRCCS E. Medea, Associazione La Nostra Famiglia (Italy), North East Research Ethics Service, NHS (UK), the Western Institutional Review Board (US), and the TREAT-NMD Global Databases Oversight Committee.

### Outcome measures

We assessed caregiver HRQL using the EuroQol EQ-5D-3L (EQ-5D) [25], a Visual Analogue Scale (VAS), and the SF-12 Health Survey (SF-12) [26]. The EQ-5D is a generic HRQL instrument encompassing five dimensions (mobility, self-care, usual activities, pain/discomfort, and anxiety/depression), each described in three levels. EQ-5D outcomes are linked to preference values, known as utilities ranging from 0 = dead to 1 = perfect health, derived from the general public. The VAS was presented as a continuous response scale, ranging from 0 = “worst imaginable health” to 1 = “best imaginable health”, measuring self-perceived HRQL.

The SF-12 is a generic HRQL instrument comprising 12 questions, each described in three to five levels. SF-12 outcomes include two composite scores, the Physical Component Summary Score (PCS) and the Mental Health Component Summary Score (MCS), as well as eight separate scores. The instrument uses norm-based scoring (mean = 50, SD = 10) and values <43 or >56 are considered significantly different from the general population [26].

In addition to the HRQL instruments, we assessed the caregiver burden using the Zarit Caregiver Burden Interview (ZBI) [27]. The ZBI contains 22 questions, each described in five levels (global score range from 0 = low burden to 88 = high burden).

To investigate the possible association between patient and caregiver HRQL, we also asked caregivers to rate their sons’ current health (categories ranging from “excellent” to “poor”) and mental status (categories ranging from “happy and interested in life” to “very unhappy”).

### Statistical analysis

We assessed mean EQ-5D utility using the recommended and most robust valuation set derived through the time-trade-off method [25] (results for other EQ-5D valuation sets presented as supplemental material online), mean VAS scores, mean global caregiver ZBI scores, and mean SF-12 PCS and MCS scores. We assessed and reported EQ-5D results for anxiety and depression separately as we hypothesized that this domain would be most influenced by the caregiver role.

We related our results to the progression of DMD by classifying patients into four groups defined first in terms of current ambulatory status and second in terms of age: (1) early ambulatory (approx. age 5–7 years), (2) late ambulatory (approx. age 8–11 years), (3) early non-ambulatory (approx. age 12–15 years), and (4) late non-ambulatory (approx. 16 years of age, or older) [8, 9].

We compared our estimates with EQ-5D and VAS general population reference data [28, 29] using Welch’s *t* tests and Welch’s analysis-of-variance models. We fitted five logistic regression models to test for differences in anxiety and depression across patients’ ambulatory status (model I), caregivers’ ratings (model II and III), and two objective measures of the caregiver burden (annual household cost burden and hours of leisure time devoted to informal care [11]) (model IV and V). Goodness-of-fit was assessed using Hosmer and Lemeshow’s test. All analyses were conducted in Stata 14.

## Results

Demographic statistics of the participating caregivers (*n* = 770) are presented in Table 1. Patients had a mean age of 14 years (median 12 years; interquartile range 9–17 years), 47 % (359 of 770) were wheelchair dependent, 16 % (126 of 770) required ventilation support, and 63 % (486 of 770) were currently taking glucocorticoids. Additional details of the study sample have been previously published [11, 23].

**Table 1:** Demographic statistics of the DMD caregivers (*n* = 770)

	*n* (proportion %)
Country of residence	
Germany	173 (22)
Italy	122 (16)
The UK	191 (25)
The US	284 (37)
Sex, female	609 (79)
Age, mean (SD) (years)	44 (8)
University degree	324 (42)
Marital status	
Married/partner	656 (85)
Separated/divorced	75 (10)
Single	30 (4)
Widowed	9 (1)
Relationship to the patient	
Parent	753 (98)
Other relative, friend, or partner	17 (2)
Current situation	
Employed	469 (61)
Unemployed	257 (33)
Retired	26 (3)
Student	12 (2)
Sick leave (>3 months)	6 (1)
Household income class^a^	
Poor	72 (9)
Middle class	615 (80)
Rich	83 (11)
Annual household cost burden^b^	
<$1000	380 (49)
$1000–$5000	170 (22)
>$5000	220 (29)
Hours of leisure time devoted to informal care (per week)	
<25	294 (38)
25–50	203 (26)
>50	273 (35)
Additional household member with DMD	55 (7)

Because of rounding, percentages might not add up to 100 % exactly

^a^Poor income class: <60 % of national median equalized household disposable income; rich income class: >200 % of national median equalized household disposable income

^b^Include non-reimbursed payments for insurance premiums, co-payments for medical and community services and medications, and out-of-pocket payments for investments (e.g., non-reimbursed payments for medical and non-medical aids and devices and investments to and reconstructions of the home)

### Prevalence of anxiety and depression

**Fig. 1:**
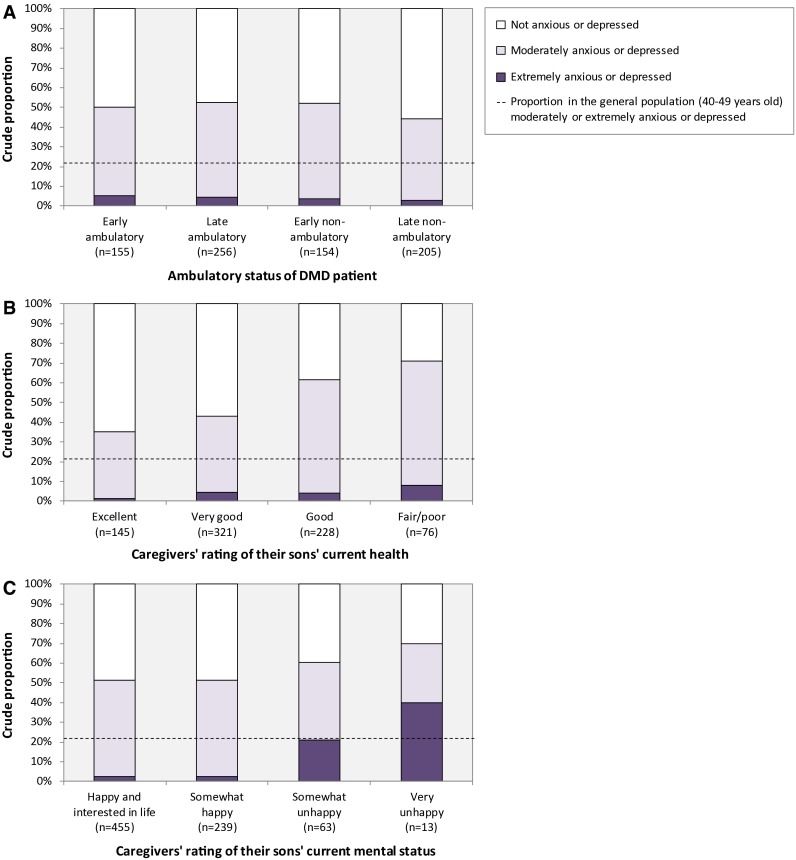
Prevalence of anxiety and depression in DMD caregivers

Half of all caregivers (383 of 770) reported being moderately or extremely anxious or depressed, significantly higher than general population reference data for individuals aged 40–49 years across all investigated strata (*p* < 0.001 for all comparison) (Fig. 1). Adjusted logistic regression results showed that anxiety and depression was strongly associated with the caregivers’ rating of patients’ health and mental status, as well as measures of objective burden (i.e., annual household cost burden and hours of leisure time devoted to informal care), but not ambulatory class (Table 2). The prevalence of anxiety and depression was comparable across countries (*p* = 0.139).

**Table 2:** Predictors of anxiety and depression in DMD caregivers

	*n*	Odds ratio (95 % CI)^a^	*p* value
Model I: patients’ ambulatory status			
Early ambulatory	155	1	
Late ambulatory	256	1.08 (0.70–1.65)	0.742
Early non-ambulatory	154	1.04 (0.64–1.70)	0.873
Late non-ambulatory	205	0.93 (0.53–1.64)	0.807
Model II: caregiver perceptions’ of patients’ health			
Excellent	145	1	
Very good	321	1.53 (1.00–2.33)	0.049
Good	228	3.85 (2.40–6.20)	<0.001
Fair/poor	76	5.87 (3.05–11.29)	<0.001
Model III: caregivers’ perception of patients’ mental status			
Happy and interested in life	455	1	
Somewhat happy	239	1.85 (1.32–2.58)	<0.001
Somewhat unhappy	63	4.67 (2.44–8.92)	<0.001
Very unhappy	13	7.22 (1.79–29.09)	0.005
Model IV: annual household cost burden			
<$1000	380	1	
$1000–$5000	170	1.43 (0.95–2.16)	0.090
>$5000	220	1.76 (1.18–2.63)	0.006
Model V: hours of leisure time devoted to informal care (per week)			
<25	294	1	
25–50	203	2.01 (1.37–2.94)	<0.001
>50	273	3.35 (2.32–4.83)	<0.001

Hosmer and Lemeshow’s test indicated good fit of the models to the data

^a^Adjusted for country, caregiver sex, caregiver age, caregiver university degree, caregiver marital status, additional household member with DMD, household income class, patient diagnosis for depression, ADHD, ASD, and OCD, patient learning disabilities, patient glucocorticoid use, and patient-caregiver relationship (parent vs. other)

### Caregiver health-related quality of life

**Fig. 2:**
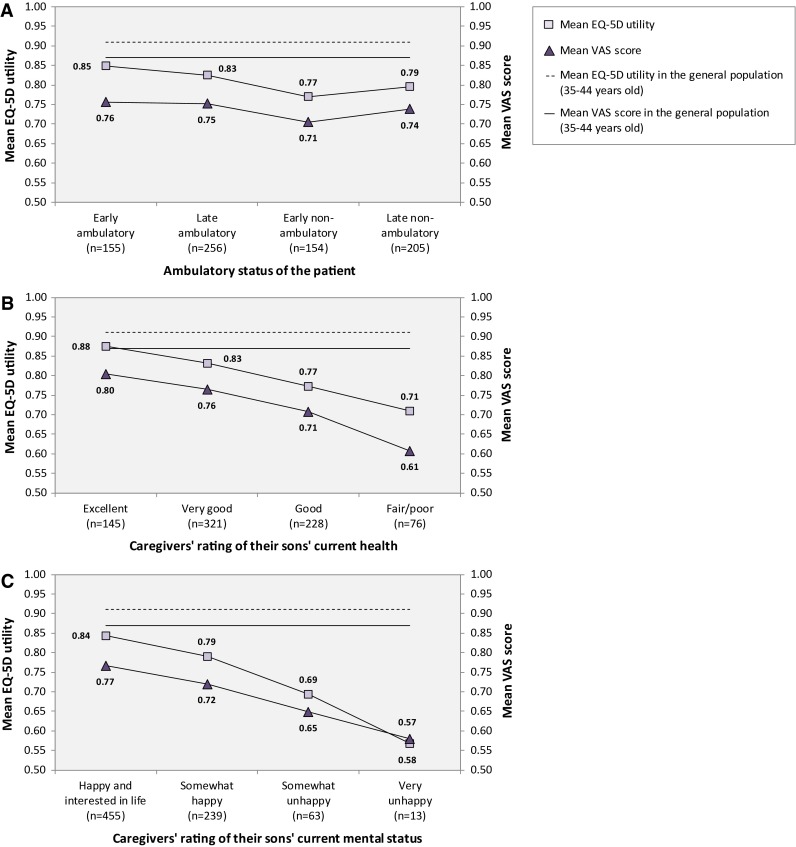
Caregiver health-related quality of life

Mean EQ-5D utilities, ranging from 0 = dead to 1 = perfect health representing general public preferences of HRQL, are presented in Fig. 2. The sex- and age-matched loss in caregiver utility in relation to the general population was estimated at between 0.09 (95 % CI 0.07–0.11) and 0.14 (0.11–0.17) across ambulatory classes, between 0.06 (0.04–0.07) and 0.18 (0.13–0.23) across caregivers’ rating of their sons’ current health, and between 0.09 (0.07–0.10) and 0.30 (0.13–0.46) across caregivers’ rating of their sons’ current mental status. Compared with general population reference data for individuals aged 40–49 years, a significantly larger proportion of DMD caregivers reported having pain or discomfort (44 vs. 33 %, *p* < 0.001) and problems performing usual activities (18 vs. 16 %, *p* = 0.006). Additional EQ-5D utility results available as supplemental material online.

Mean VAS scores, representing the caregivers’ subjective rating of their own HRQL ranging from 0 = “worst imaginable health” to 1 = “best imaginable health”, was lower than the estimated EQ-5D utilities in all strata, except for caregivers to patients rated to be very unhappy (Fig. 2). Neither mean utilities nor VAS scores were significantly different across countries.

Mean SF-12 MCS score, ranging from 0 to 100 where higher score reflects higher HRQL, was estimated at 44 (95 % CI 43–45), ranging between 44 (42–45) and 46 (45–48) across ambulatory classes, 48 (47–50) and 37 (35–40) across the caregivers’ rating of their sons’ current heath, and 46 (45–47) and 33 (26–40) across the caregivers’ rating of their sons’ current mental status. Mean PCS scores were within the normal range in all strata.

### Subjective caregiver burden

**Fig. 3:**
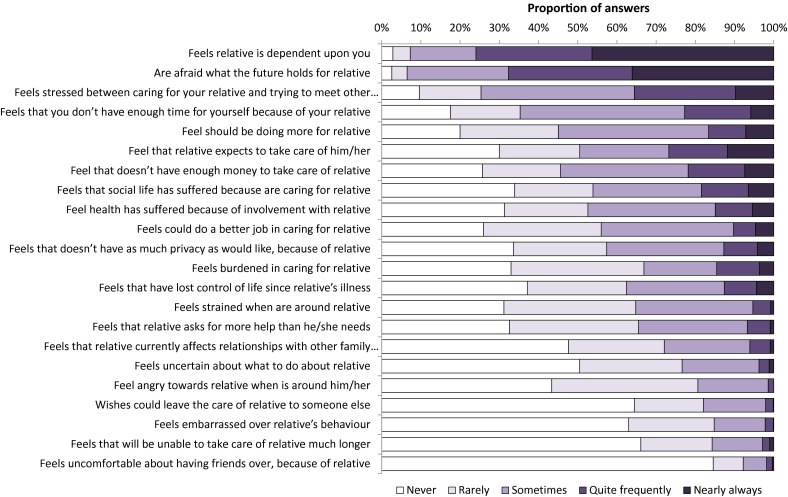
Distribution of replies from the Zarit Caregiver Burden Interview

The mean global ZBI score, ranging from 0 = low burden to 88 = high burden, was estimated at 29 (95 % CI 28–30), ranging between 25 (23–27) and 32 (30–34) across ambulatory classes, 23 (21–25) and 38 (34–41) across the caregivers’ rating of sons’ current health, and 26 (24–27) and 41 (34–48) across the caregivers’ rating of sons’ current mental status. Results from the ZBI, sorted by score (i.e., extent of caregiver burden) for each question, are presented in Fig. 3. Additional ZBI results available as supplemental material online.

## Discussion

Caregiver burden has received considerable attention in the gerontology literature during recent decades, but relatively few studies have investigated the impact on HRQL in caregivers to children with chronic diseases. Compared with adult caregivers of patients with diseases associated with advanced age, such as dementia or Parkinson’s, parents raising a child with a chronic illness may face even greater challenges as they normally live together and have no choice but to fully take on the caregiver role [1]. In addition, as a result of improved survival in many childhood diseases, including DMD, the duration of informal caregiving has increased considerably, in some indications from years to several decades, with increased morbidity and care needs in later stages of the patient's life [30, 31]. For incurable, terminal, progressive conditions, awareness of the devastating disease trajectory would also be expected to have serious adverse effects on caregiver mental well-being, and for genetic diseases, there may also be aspects of guilt.

The objective of this study was to investigate the subjective caregiver burden associated with DMD. Overall, half of all DMD caregivers in our sample indicated being moderately or extremely anxious or depressed, and we also found the mental health summary score from the SF-12 to be significantly lower than general population reference data. These result confirms previous findings of elevated risks of depression and distress reported in caregivers to children with DMD and Becker muscular dystrophy [15–17], type 1 diabetes [32], and autism [33], as well as parenting stress in pediatric chronic illnesses [34]. We found the prevalence of anxiety and depression to be comparable over the course of disease progression but strongly associated with the health and mental state of the patient as perceived by the caregiver. Specifically, caregivers to patients in fair/poor compared to excellent health had a sixfold risk increase of anxiety and depression. Comparing patients perceived as very unhappy and happy, we noted a sevenfold risk increase. These data suggest that it may be relevant to screen for anxiety and depression in caregivers to patients with DMD, and that patients’ health and mental status may be helpful predictors of caregiver distress.

To our knowledge, only one study has previously estimated HRQL in DMD caregivers using the EQ-5D, and in contrast to our findings, this study found the prevalence of anxiety and depression comparable to the general population [20]. Possible reasons for this discrepancy include differences in sample size and patient demographics, as the previous study was based on parents to 57 adult patients with DMD (mean age 27 years).

We found overall caregiver HRQL, as measured by EQ-5D utilities representing general population preferences, to be markedly impaired and closely associated with patient health and mental status, but not ambulatory class. Specifically, the mean caregiver utility was recorded at 0.81, significantly lower than the only previous report in DMD (0.87, *p* < 0.001) [20], and we estimated the mean age- and sex-matched disutility at between 0.06 and 0.30 across the investigated strata (mean in the pooled sample was 0.11). This implies that the loss in HRQL among DMD caregivers was similar to or greater than published disutility estimates for patients suffering from very serious and sometimes rapidly fatal diseases, including lung cancer and schizophrenia (0.11), systemic lupus erythematosus (0.08), and epilepsy (0.07) [35]. Given how insensitive the EQ-5D appears to be to capture impairment in HRQL in many conditions (e.g., breast and prostate cancer, asthma, and myocardial infarction, all with disutilities below the minimally important difference threshold of 0.074) [35, 36], the considerable loss in utility observed in our study is both surprising and noteworthy, and captures the exquisite stresses associated with caring for a child with a chronically disabling and progressive condition, with an invariably fatal outcome. The small change in utility and VAS scores across ambulatory classes indicates that caregivers may find ways to learn to cope with the disease and the increasing levels of disability and morbidity associated with the progression of DMD, and adjust their perception of their own HRQL over time. As shown in Fig. 2, the mean utility and VAS scores were in fact slightly higher among caregivers to late compared to early non-ambulatory patients (although the differences were not statistically different, *p* = 0.254 and 0.113, respectively), possibly related to additional assistance from, e.g., nurses and other healthcare practitioners when patients become more disabled. Comparing results from the SF-12 and the EQ-5D, it is also worth noting that the former indicated normal physical health, whereas EQ-5D domains relating to pain and discomfort and usual activities were found to be significantly impaired in relation to the general population. These inconsistent results may be explained by differences in the design and scoring of the two instruments, but warrants further research, in particular considering the recognized notion that without mental health there can be no true physical health [37].

Kenneson et al. show that employment outside of the home is a predictor of stress in caregivers to boys with DMD and Becker muscular dystrophy [16]. In line with this finding, across countries, we have previously reported that between 27 and 49 % of caregivers in our sample reduced their working hours or stopped working completely due to their son’s DMD, and we have also estimated the mean number of hours of leisure time devoted to informal caregiving per week at between 33 and 44 [11]. Still, in the present study, depending on their rating of patient well-being, between 12 and 40 % of caregivers replied that they frequently or always felt that they should be doing more for their son. We also found that between 57 and 86 % of caregivers frequently or always felt worried about the future of their child, 26 and 69 % were stressed between the demands of caring for the relative and trying to meet other responsibilities for family or work, and 17 and 62 % that they did not have enough money to take care of their son. Together with our findings that a high annual household cost burden and >25 h of leisure time devoted to informal care per week is associated with anxiety and depression, these data emphasize that lack of resources (i.e., time and money) is an important source of distress in DMD caregivers.

We estimated the mean global ZBI score at 29, ranging from 23 to 41 across investigated strata, which may be compared with estimates from studies in other diseases, for example neuromuscular diseases in general (23) [13], irritable bowel syndrome (22) [38], Alzheimer’s disease (29) [39], obsessive–compulsive disorder (29) [40], and Parkinson’s disease (24) [41]. Although these findings indicate that caring for a patient with DMD is burdensome, it is difficult to further interpret the results as there is no link between ZBI scores and subjective caregiver burden or well-being. For example, to what extent are caregivers burdened by the feeling that their sons are dependent on them, or that they feel embarrassed over their sons’ behavior, or by not having as much privacy as they would like? This would be expected to vary across caregivers, by age, sex, cultural setting, and a range of other factors. In other words, the ZBI scoring algorithm does not take into account that the questions and response categories included in the instrument may have a different impact on the self-perceived burden of each caregiver.

Despite the negative impact on well-being, there is also evidence that being a caregiver to an individual with DMD can be a positive, rewarding experience. For example, Pangalila et al. found that 95 % of DMD caregivers in their sample regarded caring as enjoyable, and Magliano et al. found that 88 % of caregivers to patients with muscle dystrophies had got something positive out of the situation [18, 20]. We did not specifically measure positive aspects of caregiving in DMD, but we found some support for these previous results in the ZBI, where 82 % of caregivers never/rarely wished that they could leave the care of their child to someone else, indicative of the devotion with which these caregivers take on their responsibilities, despite the levels of stress and demand.

Although the observational nature of our data prevents us from drawing conclusions of causality, our results have several implications for health policy. First, given the association between number of hours spent providing informal care and caregiver mental health, respite care and similar initiatives is urgently needed to help reduce the family burden and improve caregiver well-being. Second, our results suggest that many families caring for a person with DMD require increased financial support to help shoulder the considerable cost burden associated with the disease, of particular importance given the association between annual household costs and caregiver anxiety and depression demonstrated in this study. It should be noted however that some family caregivers may increase their spending because they are feeling depressed or anxious (to help improve the situation of their child), not vice versa. Third, psychosocial support for caregivers and families must be improved, especially given the poor coverage of these types of services among patients as described in our previous work [23].

Primary strengths of our study include a multinational sample, comprising patients with DMD only (as compared to mixed cohorts of neuromuscular disorders), of sufficient power to enable meaningful stratification across both disease stage and patients’ perceived health and mental status. We found patient clinical data to be characteristic for the different ambulatory classes and the distribution of age similar for responders and non-responders (not reported), indicating that any discrepancy between our patient sample and the general DMD population would be limited. We chose to recruit patients via the TREAT-NMD national DMD registries, which accept registration on a voluntary basis from patients and families with a mutation-confirmed diagnosis of DMD. Although participation in the registries is family initiated, and therefore more likely to be sought by motivated families, the registry-based approach allowed an unbiased sample to be obtained with respect to attendance at any one clinic or restriction to any one domain with potentially different care practices. Our questionnaire was returned with a mean response rate of 42 % which is comparable to other surveys sent out via this kind of route: indeed it is also worth pointing out that the response rate among those who actually received a study invitation would be expected to be notably higher as a result of, e.g., lost invitations due to recent changes to email addresses and spam filters. This also means that our burden estimates should be viewed as conservative, as very distressed caregivers may not have had time nor energy to participate. Despite some limitations in the methodology therefore we believe that this strategy has given us the best possible chance of a generalizable view of burden of care in DMD and an unprecedented glimpse into a large sample size in a rare disease.

## Conclusions

We show that caring for a person with DMD can be associated with a substantial burden and markedly impaired HRQL. Our data underscore the need for healthcare practitioners involved in the medical management of DMD to also pay attention to caregiver mental health, in particular when the health and mental status of the patient is perceived as poor. Our findings emphasize the need for a holistic approach to family mental health in the context of chronic childhood disease.

## Electronic supplementary material

Below is the link to the electronic supplementary material.

Supplementary material 1 (DOCX 258 kb)
