# Supplementary material for: Quantifying the burden of caregiving in Duchenne muscular dystrophy
Source: J Neurol. 2016 Mar 10;263:906–15. doi: 10.1007/s00415-016-8080-9 (PMC4859858; doi:10.1007/s00415-016-8080-9)
Supplement: Supplementary file 1 — Supplementary material 1 (DOCX 258 kb) [file 415_2016_8080_MOESM1_ESM.docx]

**Supplemental material**

**Additional EQ-5D-3L results**

**eTable 1: EQ-5D-3L utilities, by valuation set**

|  | **Valuation set population** | | | | |
| --- | --- | --- | --- | --- | --- |
|  | **German** | **Italian** | **UK** | **US** | **Mixed** |
| Patients’ ambulatory class |  |  |  |  |  |
| Early ambulatory | 0.94 (0.12) | 0.92 (0.08) | 0.85 (0.17) | 0.87 (0.13) | 0.89 (0.14) |
| Late ambulatory | 0.92 (0.14) | 0.90 (0.10) | 0.83 (0.19) | 0.86 (0.14) | 0.87 (0.15) |
| Early non-ambulatory | 0.88 (0.15) | 0.88 (0.10) | 0.77 (0.20) | 0.83 (0.14) | 0.84 (0.15) |
| Late non-ambulatory | 0.89 (0.16) | 0.89 (0.11) | 0.79 (0.20) | 0.84 (0.15) | 0.85 (0.17) |
| Patients' health status |  |  |  |  |  |
| Excellent | 0.95 (0.08) | 0.93 (0.07) | 0.88 (0.14) | 0.90 (0.11) | 0.90 (0.12) |
| Very good | 0.92 (0.14) | 0.91 (0.10) | 0.83 (0.19) | 0.87 (0.14) | 0.87 (0.15) |
| Good | 0.89 (0.16) | 0.88 (0.10) | 0.77 (0.20) | 0.82 (0.13) | 0.84 (0.16) |
| Fair/poor | 0.84 (0.19) | 0.84 (0.11) | 0.71 (0.23) | 0.78 (0.16) | 0.79 (0.19) |
| Patients' mental status |  |  |  |  |  |
| Happy and interested in life | 0.93 (0.13) | 0.91 (0.09) | 0.84 (0.18) | 0.87 (0.13) | 0.89 (0.14) |
| Somewhat happy | 0.90 (0.15) | 0.88 (0.10) | 0.79 (0.19) | 0.83 (0.13) | 0.85 (0.16) |
| Somewhat unhappy | 0.84 (0.18) | 0.84 (0.09) | 0.69 (0.22) | 0.77 (0.15) | 0.77 (0.15) |
| Very unhappy | 0.74 (0.26) | 0.78 (0.13) | 0.57 (0.29) | 0.69 (0.19) | 0.66 (0.28) |

*Note: n=770. Data presented as mean (SD).*

*Reference: EQ-5D value sets: Inventory, comparative review and user guide. Eds. Szende*

*A, Oppe M, Devlin N. EuroQoL Group Monographs Volume 2. Springer, 2006.*

**Additional Zarit Caregiver Burden Interview (ZBI) results**

**eFigure 1: Zarit Caregiver Burden Interview global scores**

*Note: The mean global ZBI score range from 0 to 88, where a higher score implies a larger caregiver burden.*

Results from the ZBI, stratified by the caregivers’ rating of their sons’ current health and mental status and sorted by score (i.e., extent of caregiver burden) for each question as found in the pooled analysis, are presented in below. Due to comparable results, no stratified analysis of replies was performed by ambulatory class.

**eFigure 2: Zarit Caregiver Burden Interview replies for patients rated to be in excellent health**

**eFigure 3: Zarit Caregiver Burden Interview replies for patients rated to be in very good health**

**eFigure 4: Zarit Caregiver Burden Interview replies for patients rated to be in good health**

**eFigure 5: Zarit Caregiver Burden Interview replies for patients rated to be in fair/poor health**

**eFigure 6: Zarit Caregiver Burden Interview replies for patients rated to be happy and interested in life**

**eFigure 7: Zarit Caregiver Burden Interview replies for patients rated to be somewhat happy**

**eFigure 8: Zarit Caregiver Burden Interview replies for patients rated to be somewhat unhappy**

**eFigure 9: Zarit Caregiver Burden Interview replies for patients rated to be very unhappy**
